# Structural Behavior Analysis of a Bone-Scaffold System According to the Elastic Modulus of Bone Cement and Pore Size in the Proximal Femur

**DOI:** 10.3390/jfb17050256

**Published:** 2026-05-20

**Authors:** Han Kyu Lee, Jun Won Choi, Jung Jin Kim

**Affiliations:** Department of Mechanical Engineering, Keimyung University, 1095 Dalgubeol-daero, Dalseo-gu, Daegu 42601, Republic of Korea; 1115172@stu.kmu.ac.kr (H.K.L.); 1005925@stu.kmu.ac.kr (J.W.C.)

**Keywords:** bone cement, bone scaffold, finite element analysis, strain energy, proximal femur

## Abstract

Bone scaffolds are porous artificial structures that replace damaged bone tissue and promote bone regeneration. In clinical settings, bone cement is used to provide initial fixation stability between the bone scaffold and surrounding bone tissue. To analyze the performance of bone scaffolds more accurately, the cement mantle should be considered. This study considers the cement mantle between the bone scaffold and surrounding bone tissue and the structural behavior according to variations in the elastic modulus of the cement mantle and the pore size of the bone scaffold. The results showed that the cement mantle energy ratio increased with increasing pore size, particularly in the femoral head and intertrochanteric region. In the femoral head with a pore size of 1.50 mm, increasing the cement mantle elastic modulus from 7 to 24 GPa reduced the mean strain energy within the bone scaffold from 3.79 μJ to 2.51 μJ, corresponding to a decrease of approximately 33.8%. These findings suggest that as cement mantle stiffness increases, external loads may not be sufficiently transferred to the bone scaffold interior, and the proportion of the load borne by the cement mantle may increase. In the femoral neck, the cement mantle energy ratio also increased with increasing pore size; however, the magnitude of this change was more limited than that in the other regions of interest. These findings highlight the mechanical importance of the cement mantle and suggest that both cement stiffness and scaffold pore size should be jointly considered to ensure appropriate load sharing for bone regeneration.

## 1. Introduction

Bone scaffolds have been widely investigated as implantable structures for supporting bone defect repair and regeneration. Their porous architecture provides space for cell attachment and proliferation, which is essential for tissue formation [1]. These pores also facilitate the transport of nutrients and oxygen, contributing to an environment favorable for new bone formation [2,3]. In addition, biodegradable bone scaffolds can gradually resorb in the body over time, reducing the need for additional removal surgery [4,5]. Owing to these characteristics, bone scaffolds have attracted considerable attention as effective therapeutic strategies in the field of biomechanics.

With the growing interest in bone scaffolds, research in this area has been actively conducted. For example, studies have investigated the effects of pore architecture and the material properties of bone scaffolds on their structural behavior [6]. In addition, studies have analyzed the structural behavior of patient-specific bone scaffolds implanted at femoral defect sites [7]. Furthermore, research has been conducted to optimize the implantation angle of patient-specific bone scaffolds [8]. However, previous studies predominantly focused on the structural behavior and functional performance of bone scaffolds. Consequently, the cement mantles used in clinical settings have not been sufficiently considered. In particular, these previous studies evaluated the scaffold itself or assumed direct contact between the bone and scaffold; the mechanical role of a cement mantle located between the scaffold and surrounding bone tissue has rarely been explicitly analyzed [9,10]. Therefore, the influence of the cement mantle on load transfer and strain energy distribution in a bone–cement–scaffold system remains unclear.

Bone cement is a medical material used to fill bone defect sites and bridge the gap between the bone scaffold and surrounding bone tissue [11,12]. It plays an important role in stabilizing bone scaffolds immediately after surgery, thereby providing structural stability [13,14]. When external loading is applied, the load transferred to the bone scaffold is transmitted through the cement mantle. The mechanical properties of the cement mantle may influence load transfer and stress distribution throughout the overall structure [15,16]. Therefore, the cement mantle should be considered to represent clinical conditions more accurately. Unlike scaffold-only models, a bone–cement–scaffold model can better represent the clinical fixation environment by considering the cement mantle as an intermediate load-transfer layer.

The mechanical properties of the cement mantle vary depending on the material composition, mixing ratio, and curing conditions [17,18,19]. Experimental studies on calcium-phosphate-based bone cement have confirmed that the elastic modulus can vary according to the crystalline phase formed, porosity, and mixing ratio [20,21,22]. The elastic modulus of the cement layer has been reported to vary over a wide range depending on these conditions. This indicates that the cement layer does not possess a single set of material properties in clinical practice and suggests that variations in its material properties may alter its structural behavior. Therefore, quantitatively investigating the effect of differences in the elastic modulus of the cement mantle on load transfer and the structural behavior of the bone scaffold and surrounding bone tissue is vital. However, the combined effects of cement mantle stiffness and scaffold pore size on the structural behavior of the proximal femur have not been sufficiently clarified.

The objective of this study was to quantitatively test whether changes in the elastic modulus of the cement mantle and the pore size of the bone scaffold alter load transfer and strain energy distribution between the bone scaffold and cement mantle. Through this analysis, this study aimed to evaluate the mechanical load-transfer role of the cement mantle and its necessity for structural behavior analysis during bone scaffold implantation. To this end, the scaffold was implanted in the femoral head, which is a representative site affected by avascular necrosis, and in the femoral neck and intertrochanteric region, which are susceptible to osteoporotic fractures owing to their relatively low bone density [23,24,25]. At each implantation site, a cement mantle was modeled between the bone scaffold and the surrounding bone tissue. In addition, bone scaffolds with different pore sizes were used to compare the structural behavior according to changes in the elastic modulus of the cement mantle. A finite element analysis was performed under daily load conditions, and the resulting structural behavior associated with variations in the elastic modulus of the cement mantle was analyzed. The structural behavior was quantitatively analyzed using the mean strain energy and cement–mantle energy ratio.

## 2. Methodology

**Figure 1:**
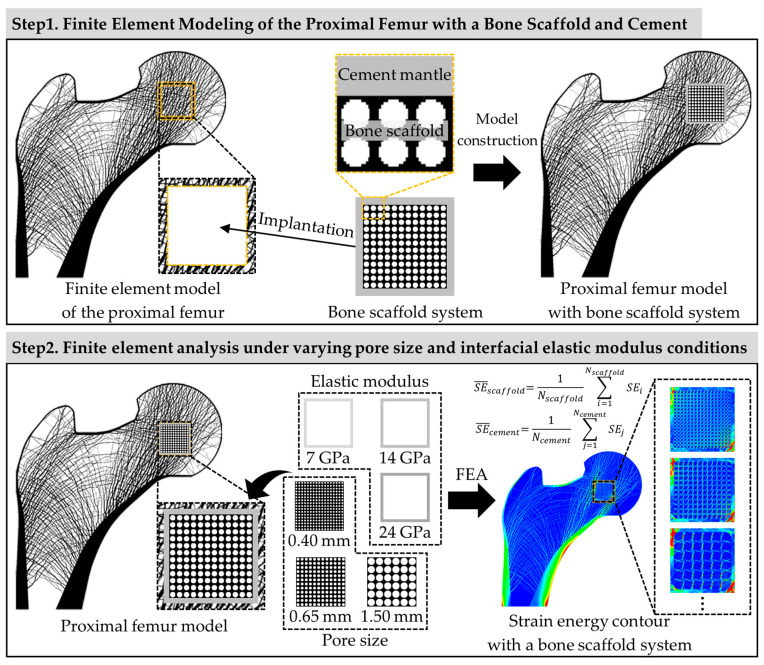
Schematic of progress for the structural behavior analysis of the bone-scaffold system.

This study quantitatively analyzed the structural behavior of the cement mantle and bone scaffold regions according to variations in the elastic modulus of the cement mantle and the pore size of the bone scaffold. This study comprised two steps (Figure 1). First, a finite element model including the proximal femur and bone scaffold was constructed, and a cement mantle was modeled between the bone scaffold and the surrounding bone tissue. In the second step, a finite element analysis was performed under daily load conditions, with pore size and the elastic modulus of the cement mantle as variables, and the structural behavior under each condition was analyzed from the perspective of strain energy.

### 2.1. Construction of the Femoral Finite Element Model

**Figure 2:**
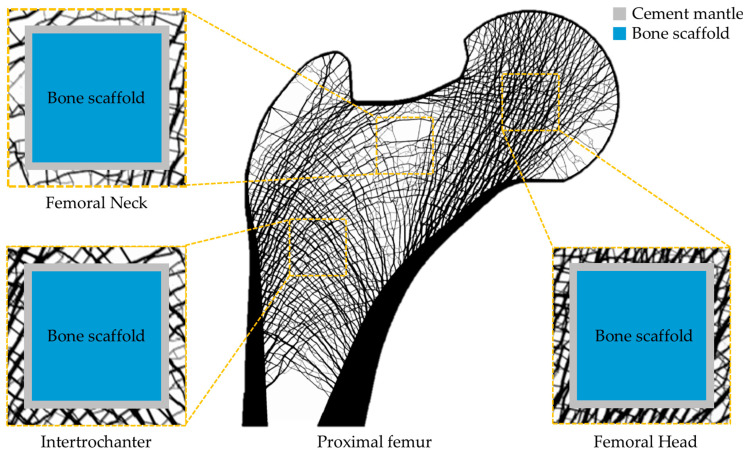
Finite element model of the proximal femur with the scaffold system and regions of interest.

In this study, a finite element model of the proximal femur was constructed using a pixel-based finite-element-modeling technique. In pixel-based finite element modeling, each pixel has a square shape with four vertices, and each vertex is converted into a node. Accordingly, each pixel is represented as a bilinear Lagrange 4-node element, which was assembled to form a finite element model (FEM) [26,27,28]. The original resolution of the image data used in this study was 600 μm, and each element was subdivided into 12 × 12 pixels to establish a more refined analysis model. Through this subdivision process, the final element size was set to approximately 50 μm. This approach enabled the image data containing various types of bone mineral density information to be reflected more accurately in the analysis model. One of the main features of this modeling approach is that the FEM can be generated directly from the image data [29,30]. This model has been widely used in previous studies to analyze the structural behavior of the proximal femur. The model includes the characteristic trabecular bone patterns observed in the human proximal femur (Figure 2). These structures include the primary tensile, primary compressive, secondary tensile, and secondary compressive groups. A proximal femur model reflecting these structural characteristics has also been used in previous studies as an analytical model to evaluate the structural behavior of the proximal femur [28,31,32,33]. Although the proximal femur is a three-dimensional anatomical structure, a two-dimensional coronal-plane model was used as a proof-of-concept numerical model in this study. The coronal plane was selected because it captures the major anatomical regions and characteristic trabecular patterns of the proximal femur, including the primary tensile, primary compressive, secondary tensile, and secondary compressive groups (Figure 2). This approach has been used in previous proximal femur finite element studies and allows efficient comparison of the relative effects of cement mantle elastic modulus and scaffold pore size under identical loading and boundary conditions.

The material properties of the bone elements were assigned based on the density value of each element. The normalized density value of each element was defined as $\rho_{i}$, where 0 < $\rho_{i}$ ≤ 1. The Young’s modulus of each bone element was determined using a density–elastic modulus relationship adopted from previous studies [34]. This relationship was originally derived from experimental data and has been widely used in finite element analyses of bone structures. To apply this relationship at the continuum level, the original density–elastic modulus equation was rescaled according to the normalized density value used in this study. Therefore, the Young’s modulus of the i-th bone element was assigned using Equation (1). A constant Poisson’s ratio of 0.30 was assigned to all bone elements.

(1) $$E_{i}=0.3044{\left(2\rho_{i}\right)}^{1.49}E_{0}\;if\;\rho_{i}\leq 0.84\;E_{i}=0.1908{\left(2\rho_{i}\right)}^{2.39}E_{0}\;if\;\rho_{i}>0.84$$

where $\rho_{i}$ represents the bone density of the ith finite element (i.e., $0<\rho_{i}\leq 1$), and $E_{i}$ is the elastic modulus assigned to the i–th finite element. A constant Poisson’s ratio of 0.3 was assigned to all bone elements. $E_{0}$ denotes the Young’s modulus of the corresponding bone tissue type, with values of 22.5 GPa for cortical bone tissue and 15 GPa for trabecular bone tissue.

### 2.2. Construction of the Bone Scaffold Model

**Figure 3:**
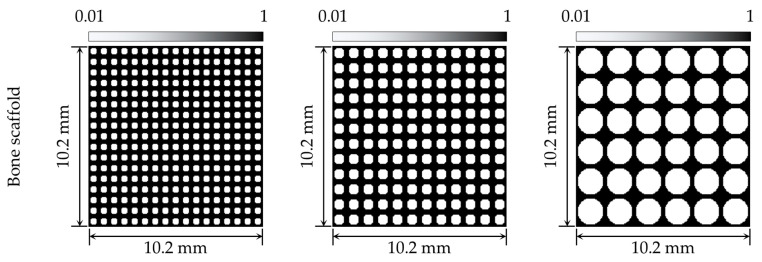
Bone scaffold according to pore size.

The bone scaffold has a periodically repeating pore architecture. Various pore architectures have been proposed for this purpose. Among these, circular pore architectures have been reported to exhibit relatively uniform load-transfer behavior [6]. In this study, a circular pore architecture was adopted to alleviate the local stress concentration and provide stable structural behavior. In addition, to compare the differences in structural behavior according to pore size, bone scaffolds with three different pore sizes were constructed (Figure 3). The pore sizes were set to 400 μm, 650 μm, and 1500 μm, respectively. The corresponding porosities were approximately 31.2%, 38.1%, and 60.2%, respectively. The thickness of the structure forming the pores was set to 100 μm. The overall size of the bone scaffold was set to 10.2 mm × 10.2 mm, considering a critical-size defect, which is difficult to heal through the natural bone regeneration process [35]. These design parameters, including pore size, strut thickness, and scaffold size, were selected based on values reported in previous scaffold studies and were used to evaluate the structural response of the scaffold system according to changes in pore size [6,8]. The resulting bone scaffold was discretized into 204 × 204 elements for finite element analysis. All elements constituting the bone scaffold model were defined as two-dimensional square elements with a size of 50 μm. In this study, the scaffold material was assumed to have the effective mechanical properties of a hydroxyapatite (HAp)-based biomaterial, rather than those of a fully dense HAp scaffold. Accordingly, the Young’s modulus and Poisson’s ratio of the scaffold were set to 13 GPa and 0.3, respectively [6,7,36].

### 2.3. Construction of the Cement Mantle

In clinical environments, bone cement exists between the bone scaffold and the surrounding bone tissue. This cement mantle may influence the load-transfer process between the two structures. Therefore, in this study, a cement mantle was included in the FEM to reflect actual clinical conditions (Figure 2). If the cement mantle is excessively thick, the contact conditions between the structures may change, which can also affect the load transfer. In this study, the cement mantle thickness was set to 0.6 mm based on the spatial resolution of the original image and the minimum load-transfer distance that could be represented in the pixel-based finite element model. According to Saint-Venant’s principle, the local effect caused by an applied load tends to become more uniformly distributed after a sufficient distance from the loading region [37,38]. The original low-resolution image used in this study had a pixel size of 600 μm, which represents the minimum distance at which the applied load and load-transfer path can be distinguished in the image-based model. Therefore, 600 μm was considered the minimum distance required for the load to become uniformly distributed before being transferred through the cement mantle to the scaffold region. Accordingly, a cement mantle thickness of 0.6 mm was applied. This thickness corresponds to 12 elements in the refined finite element model with a 50 μm element size. In addition, the elastic modulus values of the cement mantle were defined based on experimental data reported for three types of calcium phosphate cement (CPC): monetite, apatite, and brushite [39,40]. The reported values were 7.1 ± 1.0, 13.5 ± 1.6, and 24.3 ± 2.3 GPa, respectively, and the corresponding mean values of 7, 14, and 24 GPa were applied in this study (Table 1).

### 2.4. Implantation Sites of the Bone Scaffold

In this study, the femoral head, femoral neck, and intertrochanteric region within the proximal femur were selected as implantation sites for the bone scaffold. The femoral head is a region in which bone diseases, such as avascular necrosis, are likely to occur; it is also a representative region in which treatment using a bone scaffold is applied when localized bone defects are present [40]. In addition, the femoral neck and intertrochanteric regions have lower bone densities than other regions of the femur. Because of these characteristics, osteoporotic fractures frequently occur in these regions [41,42]. Considering these anatomical features, this study assumed that localized bone defects existed in these three regions and constructed finite element models in which the bone scaffold was implanted at each corresponding site (Figure 2). Based on these models, the structural behavior of the bone scaffold and cement mantle under external loading was analyzed.

### 2.5. Finite Element Analysis Conditions

**Figure 4:**
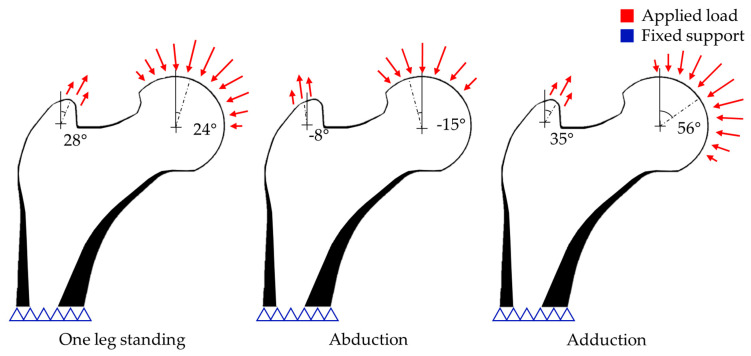
Daily loading conditions: one−leg standing, abduction, and adduction. Red arrows indicate the applied load directions, and blue triangles indicate the fixed supports.

Daily load conditions were applied in this study, with the load ratios for the single-leg stance, abduction, and adduction set at 60%, 20%, and 20%, respectively (Figure 4). These daily load conditions have been repeatedly used in previous studies on femoral loading, and their validity has been verified [43,44,45]. In addition, a fully constrained boundary condition was applied to the bottom surface of the model for the structural analysis.

### 2.6. Evaluation of Structural Behavior

In this study, the strain energy generated in the bone cement and bone-scaffold regions was calculated to quantitatively evaluate the structural behavior in each region. The strain energy represents the mechanical response to external loading and can be used to analyze the structural behavior between the bone cement and the bone scaffold [44,46]. In particular, to compare the relative structural behavior of each region, the mean strain energy was calculated by dividing the strain energy of the elements included in each region by the total number of elements in that region. This metric was used to quantitatively compare the mean energy distribution characteristics within the bone cement and bone scaffold regions (Equations (1) and (2)) [6,46]. In addition, the strain–energy ratio of the cement mantle was calculated to quantitatively evaluate the load-sharing characteristics between the cement mantle and bone scaffold. This metric represents the proportion of the total strain energy accounted for by the cement mantle (Equation (3)) and can be used to evaluate which structural component primarily carries and transfers the load within the system:

(2) $$SE_{scaffold}=\frac{1}{N_{scaffold}}\overset{N_{scaffold}}{\underset{i=1}{\sum }}SE_{i}$$

(3) $$SE_{cement}=\frac{1}{N_{cement}}\overset{N_{cement}}{\underset{j=1}{\sum }}SE_{j}$$

(4) $$R_{cement}=\frac{SE_{cement}}{SE_{cement}+SE_{scaffold}}$$

where $SE_{scaffold}$ and $SE_{cement}$ denote the mean strain energies calculated for the bone-scaffold and cement-layer regions, respectively. Here $SE_{i}$ is the strain energy of the i-th element included in the scaffold region, $SE_{j}$ is the strain energy of the j-th element included in the cement region, and $N_{scaffold}$ and $N_{cement}$ are the total numbers of elements in the bone scaffold and cement layer regions, respectively. Furthermore, $R_{cement}$ denotes the ratio of the mean strain energy of the cement layer to the sum of the mean strain energies of the two regions.

All finite element analyses were performed on a workstation equipped with an Intel Core™ i9-12900K processor (3.20 GHz) and 64 GB RAM, and ANSYS 2024 R2 (ANSYS Inc., Canonsburg, PA, USA) was used for the analyses.

## 3. Results

**Figure 5:**
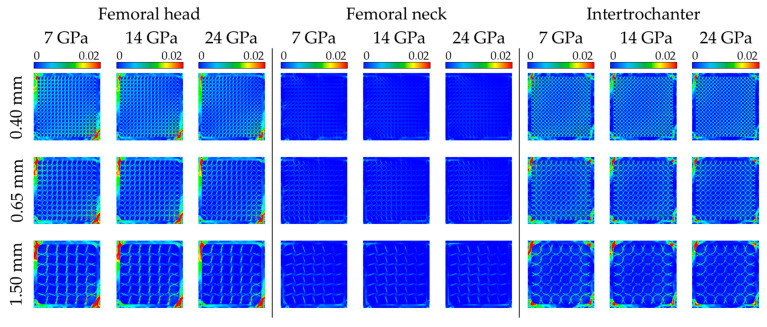
Structural behavior of the bone scaffold and cement mantle according to cement mantle elastic modulus, pore size, and implantation site (unit: μJ). The contour scale was uniformly applied to all cases for visual comparison of the strain energy distribution; quantitative comparisons were based on the mean strain energy values summarized in Table 2, Table 3 and Table 4.

Figure 5 shows the structural behavior within the bone scaffold and cement mantle according to variations in the pore size and elastic modulus of the cement mantle. Under all the pore size conditions, the mean strain energy within the bone scaffold tended to decrease as the elastic modulus of the cement mantle increased. This trend was commonly observed in the femoral head, femoral neck, and intertrochanteric regions, and became more pronounced with increasing pore size across different elastic modulus conditions. In addition, the cement–mantle energy ratio showed different patterns depending on the region of interest, and a particularly clear increasing trend with increasing pore size was observed in the femoral head and intertrochanteric region.

As the pore size of the femoral head increased, the strain energy generated in the cement mantle also increased, whereas the strain energy within the bone scaffold tended to decrease (Table 2). Specifically, when the elastic modulus of the cement mantle was 7 GPa, the strain energy in the cement mantle increased from 4.55 μJ to 6.83 μJ as the pore size increased from 0.40 mm to 1.50 mm, corresponding to an increase of approximately 50.1%. By contrast, the strain energy within the bone scaffold decreased from 4.22 μJ to 3.79 μJ, corresponding to a decrease of approximately 10.2%. In addition, the total strain energy increased from 8.77 μJ to 10.62 μJ, corresponding to an increase of approximately 21.1%, and the cement mantle energy ratio increased from 0.52 to 0.64. A similar trend was observed under cement mantle elastic modulus conditions of 14 and 24 GPa. Under the 14 GPa condition, the strain energy in the cement mantle increased from 4.73 μJ to 6.81 μJ (an increase of approximately 44.0%), whereas the energy within the bone scaffold decreased from 3.65 μJ to 3.05 μJ, corresponding to a decrease of approximately 16.4%. Simultaneously, the cement mantle energy ratio increased from 0.56 to 0.69. Under the 24 GPa condition, the strain energy in the cement mantle rose from 4.63 μJ to 6.47 μJ (approximately 39.7%), whereas the energy within the bone scaffold declined from 3.21 μJ to 2.51 μJ (a reduction of approximately 21.8%), and the cement mantle energy ratio increased from 0.59 to 0.72. Notably, for the same increase in pore size, the reduction in strain energy within the bone scaffold increased as the elastic modulus of the cement mantle increased. Accordingly, the increase in the cement mantle energy ratio also became more apparent.

Notably, the femoral neck showed a different trend compared to the femoral head and intertrochanteric region. In the femoral head and intertrochanteric region, the mean strain energy of the bone scaffold decreased as the pore size increased. In contrast, in the femoral neck, the mean strain energy of the bone scaffold increased with increasing pore size. In addition, the cement–mantle energy ratio in the femoral neck showed only limited changes compared with those in the other two regions. These results indicate that the femoral neck exhibits a site-specific load-sharing response within the bone–cement–scaffold system.

As the pore size increased in the femoral neck, the strain energy generated in the cement mantle tended to increase, whereas the strain energy within the bone scaffold showed either an increasing or decreasing tendency, depending on the condition. However, overall, the energy within the bone scaffold remained greater than that in the cement mantle, indicating that the structural behavior was predominantly governed by the bone scaffold (Table 3). Specifically, when the elastic modulus of the cement mantle was 7 GPa, the strain energy in the cement mantle increased from 0.66 μJ to 0.94 μJ as the pore size increased from 0.40 mm to 1.50 mm, corresponding to an increase of approximately 42.4%. By contrast, the strain energy within the bone scaffold increased from 0.72 μJ to 0.94 μJ, and the total strain energy increased from 1.38 μJ to 1.88 μJ, corresponding to an increase of approximately 36.2%. At the same time, the cement–mantle energy ratio increased only slightly from 0.48 μJ to 0.50 μJ, indicating that load sharing remained centered on the bone scaffold despite the increase in pore size. A similar trend was observed under cement mantle elastic modulus conditions of 14 and 24 GPa. Under the 14 GPa condition, the strain energy in the cement mantle increased from 0.54 μJ to 0.78 μJ, corresponding to an increase of approximately 44.4%, whereas the energy within the bone scaffold increased from 0.66 μJ to 0.83 μJ, corresponding to an increase of approximately 25.8%. In this case, the cement–mantle energy ratio increased from 0.45 μJ to 0.48 μJ. Under the 24 GPa condition, the strain energy in the cement mantle increased from 0.45 μJ to 0.67 μJ, corresponding to an increase of approximately 48.9%, whereas the energy within the bone scaffold increased from 0.61 μJ to 0.75 μJ, corresponding to an increase of approximately 23.0%, and the cement mantle energy ratio increased from 0.42 μJ to 0.47 μJ. Meanwhile, under the same pore-size conditions, the strain energy in the cement mantle tended to decrease as the elastic modulus of the cement mantle increased; accordingly, the cement mantle energy ratio also decreased. For example, under the 0.40 mm pore size condition, the strain energy in the cement mantle decreased from 0.66 μJ at 7 GPa to 0.45 μJ at 24 GPa, corresponding to a decrease of approximately 31.8%, while the cement mantle energy ratio also decreased from 0.48 μJ to 0.42 μJ.

As the pore size in the intertrochanteric region increased, the strain energy generated in the cement mantle increased, whereas that within the bone scaffold decreased (Table 4). Specifically, when the elastic modulus of the cement mantle was 7 GPa, the strain energy in the cement mantle increased from 2.55 μJ to 4.91 μJ as the pore size increased from 0.40 mm to 1.50 mm, corresponding to an increase of approximately 92.5%. By contrast, the strain energy within the bone scaffold decreased from 4.07 μJ to 3.33 μJ, corresponding to a decrease of approximately 18.2%. In addition, the total strain energy increased from 6.62 μJ to 8.24 μJ, corresponding to an increase of approximately 24.5%, and the cement mantle energy ratio increased markedly from 0.39 to 0.60. Under the 14 GPa condition, the strain energy in the cement mantle increased from 2.25 μJ to 4.22 μJ as the pore size increased from 0.40 mm to 1.50 mm, corresponding to an increase of approximately 87.6%. By contrast, the strain energy within the bone scaffold decreased from 3.69 μJ to 2.77 μJ, corresponding to a decrease of approximately 24.9%. In addition, the total strain energy increased from 5.94 μJ to 6.99 μJ, corresponding to an increase of approximately 17.7%, and the cement mantle energy ratio increased from 0.38 to 0.60. Under the 24 GPa condition, the strain energy in the cement mantle increased from 2.08 μJ to 3.79 μJ as the pore size increased from 0.40 mm to 1.50 mm, corresponding to an increase of approximately 82.2%. By contrast, the strain energy within the bone scaffold decreased from 3.42 μJ to 2.38 μJ, corresponding to a decrease of approximately 30.4%. In addition, the total strain energy increased from 5.50 μJ to 6.17 μJ, corresponding to an increase of approximately 12.2%, and the cement mantle energy ratio increased from 0.38 to 0.61. Meanwhile, under the same pore-size conditions, both the strain energy in the cement mantle and that within the bone scaffold tended to decrease as the elastic modulus of the cement mantle increased; however, the cement mantle energy ratio remained nearly unchanged or showed a slight increase.

## 4. Discussion

In this study, a proximal femur finite element model including the bone scaffold and cement mantle was constructed to evaluate the structural behavior of the scaffold system according to changes in cement mantle elastic modulus and scaffold pore size. The results showed that the cement mantle is an important component involved in load transfer within the bone scaffold system. In particular, changes in cement stiffness influenced the distribution of strain energy between the scaffold and the cement mantle [47,48,49]. This indicates that the cement mantle should not be regarded only as a fixation material but also as a mechanical load-transfer layer that affects the structural response of the implanted scaffold system. Therefore, the scientific contribution of this study is that it provides a more clinically relevant framework for evaluating scaffold implantation by considering the combined effects of cement mantle stiffness and scaffold pore size on load transfer and strain energy distribution in the proximal femur.

In the femoral head, the reduction in scaffold stiffness associated with larger pores appears to have led to load redistribution toward the relatively stiffer cement mantle [50,51]. This effect became more evident as the elastic modulus of the cement mantle increased, because the stiffness difference between the scaffold and cement mantle became larger. Therefore, in the femoral head, both scaffold pore architecture and cement mantle stiffness contributed to the shift in load sharing from the scaffold to the cement mantle.

The femoral neck exhibited a distinct mechanical response compared with the femoral head and intertrochanteric region. This difference may be attributed to the narrow geometry, local trabecular architecture, and limited load-transfer pathway of the femoral neck. The femoral neck connects the femoral head and femoral shaft and is subjected to combined axial and bending loads. In this region, the total strain energy was lower than that in the other regions. However, the change in load sharing with increasing pore size was limited. Therefore, the load did not shift markedly to the cement mantle. Instead, the remaining scaffold struts continued to participate in load bearing. As a result, mechanical energy was concentrated in the scaffold struts, leading to an increase in the mean strain energy of the scaffold. By contrast, in the femoral head and intertrochanteric region, the decrease in scaffold stiffness with increasing pore size promoted load transfer to the cement mantle, resulting in decreased scaffold strain energy and increased cement–mantle energy ratio.

In the intertrochanteric region, the structural behavior shifted from the bone scaffold to the cement mantle as the pore size increased. In particular, the cement mantle energy ratio increased from approximately 0.38 to more than 0.60 with increasing pore size, indicating that the load sharing progressively shifted from the bone scaffold to the cement mantle. Specifically, as pore size increased, the strain energy in the cement mantle increased by approximately 80–90%, whereas the strain energy within the bone scaffold decreased by approximately 20–30%. This change can be interpreted as a consequence of the reduced stiffness of the bone scaffold caused by the increase in pore size, which redistributed the load to the relatively stiffer cement mantle. Consequently, the total strain energy increased, and simultaneously, the distribution of the strain energy tended to shift toward the cement mantle. Under the same pore size conditions, both the strain energy in the cement mantle and within the bone scaffold tended to decrease as the elastic modulus of the cement mantle increased. However, the strain–energy ratio in the cement mantle remained nearly unchanged or showed a slightly increasing trend. This suggests that, although an increase in the stiffness of the cement mantle reduces the deformation of the overall structure, the pore architecture exerts a greater influence on energy sharing.

The mean strain energy within the bone scaffold and cement mantle, as well as the cement mantle energy ratio, exhibited different trends depending on pore size. As the pore size increased, the mean strain energy within the bone scaffold tended to decrease in the femoral head and intertrochanteric regions, whereas the mean strain energy within the cement mantle and the cement mantle energy ratio tended to increase. This indicates that changes in the pore size may affect the load distribution pattern and energy distribution characteristics of the bone scaffold and cement mantle [52]. However, despite these differences, a common trend was observed under all pore size conditions; as the elastic modulus of the cement mantle increased, the mean strain energy within the bone scaffold tended to decrease. Therefore, although the structural response may vary depending on changes in pore size, the elastic modulus of the cement mantle can be considered an important factor that determines the load transferred into the bone scaffold.

The results quantitatively confirm that the cement mantle plays an important role in the interaction between the bone scaffold and surrounding bone tissue. In particular, the cement mantle serves not only as a fixation layer but also as an intermediate layer involved in load transfer and energy distribution between the two structures. Although such a cement mantle is typically present between the bone tissue and bone scaffold in actual clinical environments, many previous studies have assumed direct contact between the two structures in their analyses [6,8,53]. In contrast to these previous studies, the present study explicitly included the cement mantle between the bone scaffold and surrounding bone tissue and evaluated the effects of variations in its elastic modulus on load transfer and strain energy distribution. However, the present study addressed the limitations of this conventional assumption by considering both the presence of the cement mantle and variations in its material properties. In addition, the mechanical properties of the cement mantle may directly affect its structural behavior. These findings confirm that the cement mantle should be considered not only as a fixation layer but also as an important factor in evaluating the structural behavior of scaffold-implanted proximal femurs.

The strain energy results also have biological implications for bone regeneration. From the perspective of Wolff’s law, strain energy can be interpreted as an indirect indicator of mechanical stimulation for bone remodeling and bone ingrowth. In this study, the strain energy of the bone scaffold generally decreased as the elastic modulus of the cement mantle increased. This suggests that more load was transferred to the cement mantle rather than to the scaffold. Although this load shifting may improve initial structural stability, it may reduce mechanical stimulation within the scaffold and surrounding bone tissue. This could lead to stress shielding and may negatively affect bone ingrowth or long-term bone remodeling. Therefore, the cement mantle should be considered not only as a fixation layer but also as an important factor affecting the balance between structural stability and biological stimulation in scaffold-based bone regeneration.

This study has several limitations. First, this study was conducted as a proof-of-concept numerical parametric study using a single proximal femur model; direct experimental validation was not performed. Therefore, the absolute mechanical response may differ from that under actual experimental or clinical conditions. Future studies should include experimental validation and comparison of the measured strain distribution with the finite element results. Second, the mechanical properties of the cement mantle were simplified by considering only changes in elastic modulus and evaluating only three discrete values, namely 7, 14, and 24 GPa. However, actual bone cement may exhibit more complex mechanical properties depending on its composition, mixing ratio, curing conditions, and porosity. These simplified conditions may limit the generalizability of the findings, and future studies should include sensitivity or regression-based analyses using a wider range of cement mantle elastic modulus values. Third, because this study was performed using a two-dimensional FEM, it could not fully reflect the three-dimensional geometry and load-transfer characteristics observed in the actual proximal femur and bone scaffold. Fourth, the cement mantle–bone and cement mantle–scaffold interfaces were assumed to be perfectly bonded in this study. However, actual clinical interfaces may involve frictional contact, sliding, debonding, or micromotion. Future studies should evaluate these various interface conditions to more accurately analyze load transfer within the bone–cement–scaffold system. Finally, this study did not consider tissue ingrowth into the scaffold pores during the healing process. Such tissue colonization may increase the effective stiffness of the scaffold and alter load transfer over time. Therefore, future studies should include time-dependent analyses that consider tissue ingrowth, bone remodeling, and gradual changes in scaffold stiffness.

## 5. Conclusions

In this study, the structural behavior of a bone scaffold with a cement mantle was analyzed using a proximal femur model. In addition, differences in the structural behavior according to variations in the elastic modulus of the cement mantle and the pore size of the bone scaffold were quantitatively evaluated. The patterns of structural behavior also differed with pore size. In the femoral head and intertrochanteric regions, the cement–mantle energy ratio increased markedly as the pore size increased, indicating that load sharing tended to shift from the bone scaffold to the cement mantle. By contrast, in the femoral neck, the change in the cement–mantle energy ratio remained limited despite the increase in pore size, indicating that load sharing remained predominantly centered on the bone scaffold. Despite these differences, a common trend was observed under all pore-size conditions: as the elastic modulus of the cement mantle increased, the mean strain energy within the bone scaffold decreased. Therefore, in designing bone scaffolds, it is important to consider not only the pore architecture of the bone scaffold itself but also the mechanical properties of the cement mantle to achieve load transfer characteristics appropriate for actual clinical environments. This approach may serve as a basis for the future design and evaluation of patient-specific bone scaffolds.

## Figures and Tables

**Table 1:** Summary of material properties assigned to the model components.

Model Component	Young’s Modulus (GPa)	Poisson’s Ratio
Cortical bone	22.5	0.3
Cancellous bone	15	0.3
Bone scaffold	13	0.3
Cement mantle	7, 14, 24	0.3

**Table 2:** Strain energy in the femoral head according to cement elastic modulus and pore size.

E [GPa]	Pore Size [mm]	SE_cement_ [μJ]	SE_scaffold_ [μJ]	Total	R_Cement_
7	0.40	4.55	4.22	8.77	0.52
	0.65	5.84	4.14	9.98	0.59
	1.50	6.83	3.79	10.62	0.64
14	0.40	4.73	3.65	8.38	0.56
	0.65	5.97	3.43	9.4	0.63
	1.50	6.81	3.05	9.86	0.69
24	0.40	4.63	3.21	7.84	0.59
	0.65	5.75	2.91	8.66	0.66
	1.50	6.47	2.51	8.98	0.72

**Table 3:** Strain energy in the femoral neck according to cement elastic modulus and pore size.

E [GPa]	Pore Size [mm]	SE_cement_ [μJ]	SE_scaffold_ [μJ]	Total	R_Cement_
7	0.40	0.66	0.72	1.38	0.48
	0.65	0.79	0.87	1.66	0.48
	1.50	0.94	0.94	1.88	0.50
14	0.40	0.54	0.66	1.20	0.45
	0.65	0.66	0.78	1.44	0.46
	1.50	0.78	0.83	1.61	0.48
24	0.40	0.45	0.61	1.06	0.42
	0.65	0.56	0.72	1.28	0.44
	1.50	0.67	0.75	1.42	0.47

**Table 4:** Strain energy in the intertrochanter according to cement elastic modulus and pore size.

E [GPa]	Pore Size [mm]	SE_cement_ [μJ]	SE_scaffold_ [μJ]	Total	R_Cement_
7	0.40	2.55	4.07	6.62	0.39
	0.65	3.85	4.09	7.94	0.48
	1.50	4.91	3.33	8.24	0.60
14	0.40	2.25	3.69	5.94	0.38
	0.65	3.39	3.54	6.93	0.49
	1.50	4.22	2.77	6.99	0.60
24	0.40	2.08	3.42	5.50	0.38
	0.65	3.09	3.16	6.25	0.49
	1.50	3.79	2.38	6.17	0.61

## Data Availability

The data generated and analyzed during this study are included in this article. Additional numerical output data and finite element model information are available from the corresponding author upon reasonable request.

## References

[1] Abbasi N., Hamlet S., Love R.M., Nguyen N.T. (2020). Porous Scaffolds for Bone Regeneration. J. Sci. Adv. Mater. Devices.

[2] Jiang S., Wang M., He J. (2021). A Review of Biomimetic Scaffolds for Bone Regeneration: Toward a Cell-Free Strategy. Bioeng. Transl. Med..

[3] Lee S.S., Du X., Kim I., Ferguson S.J. (2022). Scaffolds for Bone-Tissue Engineering. Matter.

[4] Dorati R., DeTrizio A., Modena T., Conti B., Benazzo F., Gastaldi G., Genta I. (2017). Biodegradable Scaffolds for Bone Regeneration Combined with Drug-Delivery Systems in Osteomyelitis Therapy. Pharmaceuticals.

[5] Zhou T., Ma P.X. (2026). Peptide-Conjugated Biodegradable Polyester Scaffolds for Bone Regeneration. Biomaterials.

[6] Choi J.W., Kim J.J. (2025). A Computational Approach to Investigate the Structural Behavior of Bone Scaffold-Implanted Proximal Femur in Routine Clinical Resolution. Int. J. Numer. Method. Biomed. Eng..

[7] Pandithevan P., Kumar G.S. (2010). Finite Element Analysis of a Personalized Femoral Scaffold with Designed Microarchitecture. Proc. Inst. Mech. Eng. Part H J. Eng. Med..

[8] Won J., Jung C., Kim J. (2025). Strategies for the Patient-Specific Implant Angle of Bone Scaffolds Using Optimization. Tissue Eng. Regen. Med..

[9] Rana M., Chaudhuri A., Biswas J.K., Karim S.I., Datta P., Karmakar S.K., Roychowdhury A. (2021). Design of Patient Specific Bone Stiffness Mimicking Scaffold. Proc. Inst. Mech. Eng. Part H J. Eng. Med..

[10] Wu P.K., Lee C.W., Sun W.H., Lin C.L. (2022). Biomechanical Analysis and Design Method for Patient-Specific Reconstructive Implants for Large Bone Defects of the Distal Lateral Femur. Biosensors.

[11] Qiu G., Huang M., Ke D., Liu J., Weir M.D., Ma T., Wang P., Oates T.W., Schneider A., Xia Y. (2022). Novel Injectable Calcium Phosphate Scaffold with Human Periodontal Ligament Stem Cell Encapsulation in Microbeads for Bone Regeneration. Front. Mater..

[12] Xia Y., Wang H., Li Y., Fu C. (2022). Engineered Bone Cement Trigger Bone Defect Regeneration. Front. Mater..

[13] Xu H.H.K., Wang P., Wang L., Bao C., Chen Q., Weir M.D., Chow L.C., Zhao L., Zhou X., Reynolds M.A. (2017). Calcium Phosphate Cements for Bone Engineering and Their Biological Properties. Bone Res..

[14] Zhu T., Ren H., Li A., Liu B., Cui C., Dong Y., Tian Y., Qiu D. (2017). Novel Bioactive Glass Based Injectable Bone Cement with Improved Osteoinductivity and Its in Vivo Evaluation. Sci. Rep..

[15] Chen J., Ma X., Li Z., Fan W., Ma L. (2025). Biomechanical Impact of Enoxaparin Sodium-Chitosan-PMMA Bone Cement in Hip Arthroplasty: A Preliminary Finite Element Analysis. Front. Surg..

[16] Mondal S., MacManus D.B., Banche-Niclot F., Vitale-Brovarone C., Fiorilli S., McCarthy H.O., Dunne N. (2024). Finite Element Analysis of Vertebroplasty in the Osteoporotic T11-L1 Vertebral Body: Effects of Bone Cement Formulation. J. Biomed. Mater. Res. Part B Appl. Biomater..

[17] Liu H., Zhang Z., Gao C., Bai Y., Liu B., Wang W., Ma Y., Saijilafu, Yang H., Li Y. (2020). Enhancing Effects of Radiopaque Agent BaSO_4_ on Mechanical and Biocompatibility Properties of Injectable Calcium Phosphate Composite Cement. Mater. Sci. Eng. C.

[18] Ambard A.J., Mueninghoff L. (2006). Calcium Phosphate Cement: Review of Mechanical and Biological Properties. J. Prosthodont..

[19] Qiao Q., Zhao Q., Wang J., Li M., Zhou H., Yang L. (2025). The Effects of Calcium Phosphate Bone Cement Preparation Parameters on Injectability and Compressive Strength for Minimally Invasive Surgery. Bioengineering.

[20] Takagi S., Chow L.C. (2001). Formation of Macropores in Calcium Phosphate Cement Implants. J. Mater. Sci. Mater. Med..

[21] Zhang J., Liu W., Schnitzler V., Tancret F., Bouler J.M. (2014). Calcium Phosphate Cements for Bone Substitution: Chemistry, Handling and Mechanical Properties. Acta Biomater..

[22] Kamitakahara M., Kato K., Umetsu M., Yoshihara K., Yoshida Y. (2025). Design of Bioresorbable Calcium Phosphate Cement with High Porosity via the Addition of Bioresorbable Polymers. J. Biomater. Appl..

[23] Kanazawa T., Ohmori T., Toda K., Ito Y. (2023). Relationship between Site-Specific Bone Mineral Density in the Proximal Femur and Instability of Proximal Femoral Fractures: A Retrospective Study. Orthop. Traumatol. Surg. Res..

[24] Bernstein D.N., Davis J.T., Fairbanks C., McWilliam-Ross K., Ring D., Sanchez H.B. (2018). Lower Bone Mineral Density Is Associated with Intertrochanteric Hip Fracture. Arch. Bone Jt. Surg..

[25] Hines J.T., Jo W.L., Cui Q., Mont M.A., Koo K.H., Cheng E.Y., Goodman S.B., Ha Y.C., Hernigou P., Jones L.C. (2021). Osteonecrosis of the Femoral Head: An Updated Review of ARCO on Pathogenesis, Staging and Treatment. J. Korean Med. Sci..

[26] Jang I.G., Kim I.Y. (2008). Computational Study of Wolff’s Law with Trabecular Architecture in the Human Proximal Femur Using Topology Optimization. J. Biomech..

[27] Jang I.G., Kim I.Y. (2010). Application of Design Space Optimization to Bone Remodeling Simulation of Trabecular Architecture in Human Proximal Femur for Higher Computational Efficiency. Finite Elem. Anal. Des..

[28] Kim J.J., Jang I.G. (2016). Image Resolution Enhancement for Healthy Weight-Bearing Bones Based on Topology Optimization. J. Biomech..

[29] Falcinelli C., Whyne C. (2020). Image-Based Finite-Element Modeling of the Human Femur. Comput. Methods Biomech. Biomed. Engin..

[30] Saillard E., Gardegaront M., Levillain A., Bermond F., Mitton D., Pialat J.B., Confavreux C., Grenier T., Follet H. (2024). Finite Element Models with Automatic Computed Tomography Bone Segmentation for Failure Load Computation. Sci. Rep..

[31] Lee Y.H., Kim Y., Kim J.J., Jang I.G. (2015). Homeostasis-Based Aging Model for Trabecular Changes and Its Correlation with Age-Matched Bone Mineral Densities and Radiographs. Eur. J. Radiol..

[32] Kim J.J., Nam J., Jang I.G. (2018). Computational Study of Estimating 3D Trabecular Bone Microstructure for the Volume of Interest from CT Scan Data. Int. J. Numer. Methods Biomed. Eng..

[33] Kim J., Kim J.J. (2022). Topology Optimization-Based Localized Bone Microstructure Reconstruction for Image Resolution Enhancement: Accuracy and Efficiency. Bioengineering.

[34] Verhulp E., van Rietbergen B., Huiskes R. (2006). Comparison of Micro-Level and Continuum-Level Voxel Models of the Proximal Femur. J. Biomech..

[35] Cooke M.N., Fisher J.P., Dean D., Rimnac C., Mikos A.G. (2003). Use of Stereolithography to Manufacture Critical-Sized 3D Biodegradable Scaffolds for Bone Ingrowth. J. Biomed. Mater. Res. Part B Appl. Biomater..

[36] Lin D., Li Q., Li W., Zhou S., Swain M.V. (2009). Design Optimization of Functionally Graded Dental Implant for Bone Remodeling. Compos. Part B Eng..

[37] Horgan C.O. (1989). Recent Developments Concerning Saint-Venant’s Principle: An Update. Appl. Mech. Rev..

[38] Horgan C.O., Knowles J.K. (1983). Recent Developments Concerning Saint-Venant’s Principle. Adv. Appl. Mech..

[39] Ajaxon I., Acciaioli A., Lionello G., Ginebra M., Öhman-mägi C., Baleani M., Persson C. (2017). Elastic Properties and Strain-to-Crack-Initiation of Calcium Phosphate Bone Cements: Revelations of a High-Resolution Measurement Technique. J. Mech. Behav. Biomed. Mater..

[40] Charrière E., Terrazzoni S., Pittet C., Mordasini P., Dutoit M., Lemaître J., Zysset P. (2001). Mechanical Characterization of Brushite and Hydroxyapatite Cements. Biomaterials.

[41] Yoon B.H., Lee Y.K., Kim S.C., Kim S.H., Ha Y.C., Koo K.H. (2013). Epidemiology of Proximal Femoral Fractures in South Korea. Arch. Osteoporos..

[42] Narayanan A., Khanchandani P., Borkar R.M., Ambati C.R., Roy A., Han X., Bhoskar R.N., Ragampeta S., Gannon F., Mysorekar V. (2017). Avascular Necrosis of Femoral Head: A Metabolomic, Biophysical, Biochemical, Electron Microscopic and Histopathological Characterization. Sci. Rep..

[43] Beaupré G.S., Orr T.E., Carter D.R. (1990). An Approach for Time-Dependent Bone Modeling and Remodeling—Application: A Preliminary Remodeling Simulation. J. Orthop. Res..

[44] Kim J., Chun B.J., Kim J.J. (2023). Quantitative Load Dependency Analysis of Local Trabecular Bone Microstructure to Understand the Spatial Characteristics in the Synthetic Proximal Femur. Biology.

[45] Beaupré G.S., Orr T.E., Carter D.R. (1990). An Approach for Time-Dependent Bone Modeling and Remodeling—Theoretical Development. J. Orthop. Res..

[46] Weinans H., Huiskes R., Grootenboer H.J. (1992). The Behavior of Adaptive Bone-Remodeling Simulation Models. J. Biomech..

[47] Haase K., Rouhi G. (2013). Prediction of Stress Shielding around an Orthopedic Screw: Using Stress and Strain Energy Density as Mechanical Stimuli. Comput. Biol. Med..

[48] Savio D., Bagno A. (2022). When the Total Hip Replacement Fails: A Review on the Stress-Shielding Effect. Processes.

[49] Kim S.M., Choi J.W., Kim J.J. (2024). Personalized Stem Length Optimization in Hip Replacement: A Microscopic Perspective on Bone—Implant Interaction. Bioengineering.

[50] Aydin M.S., Marek N., Luciani T., Mohamed-Ahmed S., Lund B., Gjerde C., Mustafa K., Suliman S., Rashad A. (2024). Impact of Porosity and Stiffness of 3D Printed Polycaprolactone Scaffolds on Osteogenic Differentiation of Human Mesenchymal Stromal Cells and Activation of Dendritic Cells. ACS Biomater. Sci. Eng..

[51] Chen S., Liao K., Yang Y., Chen H., Huang R. (2025). Influence of Porosity Gradient Distribution on Mechanical and Biological Properties of Gyroid-Based Zn-2Mg Scaffolds for Bone Tissue Engineering. Materials.

[52] Zhang C., Lyu Y., Semenova A., Li Z. (2025). Influence of Load-Bearing Angle, Structural Topology, and Porosity Gradient on the Energy Absorption Capability of TPMS-Based Scaffolds for Bone Tissue Engineering. Front. Bioeng. Biotechnol..

[53] He H., Liu Y., Dong Y., Zhao W., Wang K., Guo K., Jiao E., Xu S., Ju G., Wang P. (2025). Material Properties and Progress in Modification of Hydrogel-Based Self-Expandable Poly(Methyl Methacrylate) Bone Cement. RSC Adv..

